# Hybrid Full-Wave Analysis of Surface Acoustic Wave Devices for Accuracy and Fast Performance Prediction

**DOI:** 10.3390/mi12010005

**Published:** 2020-12-22

**Authors:** Zhenglin Chen, Qiaozhen Zhang, Sulei Fu, Xiaoyu Wang, Xiaojun Qiu, Haodong Wu

**Affiliations:** 1 School of Electronic Science and Engineering, Nanjing University, Nanjing 210093, China; zlchen1988@yeah.net (Z.C.); xjqiu@nju.edu.cn (X.Q.); 2 Mechanical and Electrical Engineering, College of Information, Shanghai Normal University, Shanghai 200234, China; 3 Key Laboratory of Advanced Materials (MOE), School of Materials Science and Engineering, Tsinghua University, Beijing 100084, China; fusulei@mail.tsinghua.edu.cn; 4 Key Laboratory of Modern Acoustics, Ministry of Education, Department of Acoustic Science and Engineering, School of Physics, Nanjing University, Nanjing 210093, China; wangxy512@163.com

**Keywords:** partial differential equations, graphics processing unit (GPU), hierarchical cascading technology, parasitic electromagnetics, finite element method, perfect match layer, COMSOL Multiphysics, surface acoustic wave (SAW) devices

## Abstract

In this paper, a hybrid full-wave analysis of surface acoustic wave (SAW) devices is proposed to achieve accurate and fast simulation. The partial differential equation (PDE) models of the physical system in question and graphics processing unit (GPU)-assisted hierarchical cascading technology (HCT) are used to calculate acoustic-electric characteristics of a SAW filter. The practical solid model of the radio frequency (RF) filter package is constructed in High Frequency Structure Simulator (HFSS) software and the parasitic electromagnetics of the entire package is considered in the design process. The PDE-based models of the two-dimensional finite element method (2D-FEM) are derived in detail and solved by the PDE module embedded in COMSOL Multiphysics. Due to the advantages of PDE-based 2D-FEM, it is universal, efficient and not restricted to handling arbitrary materials and crystal cuts, electrode shapes, and multi-layered substrate. Combining COMSOL Multiphysics with a user-friendly interface, a flexible way of modeling and mesh generation, it can greatly reduce the complicated process of modeling and physical properties definition. Based on a hybrid full-wave analysis, we present an example application of this approach on a TC-SAW ladder filter with 5° YX-cut LiNbO_3_ substrate. Numerical results and measurements were calculated for comparison, and the accuracy and efficiency of the proposed method were verified.

## 1. Introduction

Because of their high performance, small size and low cost, surface acoustic wave (SAW) devices have become key radio frequency (RF) micro electro-mechanical components in the wireless communication field. With the coming of the 5G era, SAW devices have gradually become stringent requirements on RF devices [1,2]. It promotes the emergence of a large number of new-type SAW devices, such as temperature compensated SAW (TC-SAW) devices [3,4,5], incredible high-performance SAW (I.H.P. SAW) devices [6,7,8,9], and laterally-excited bulk-wave resonators (XBARs) [10,11]. However, for such SAW devices with complicated structures, a universal method used for the accurate and fast simulation of the SAW devices calls for urgent demand.

As for practical SAW devices, especially SAW filters, hybrid full-wave analysis, including piezoelectric and electromagnetic analysis, is expected to be an effective way to simulate full-scale SAW devices [12]. Due to the consideration of multiphysics fields in the analysis process, including acoustic, electrostatic and electromagnetic fields, the hybrid full-wave analysis for SAW filter is expected to achieve higher accuracy. Since the filter is comprised of series and parallel resonators in which the frequency characteristics determine the response of the filter, the difficulty in simulation accuracy mainly depends on the simulation accuracy of the resonators.

As for numerical simulation tools, the finite element method/boundary element method (FEM/ BEM) is an effective way to calculate a regular SAW resonator with finite-length structures [13]. Unfortunately, this method requires a long time to compute the Green function, especially an SAW resonator with a multi-layered and intricate structure, and the FEM/BEM method cannot effectively work. The pure FEM method is an effective and universal method to deal with a multi-layered and intricate structure, moreover, FEM modeling in COMSOL Multiphysics offers a more convenient and flexible way to build arbitrary structures [14]. However, the pure FEM method has difficulties in rapid calculation of the behavior of SAW devices due to amount of degrees of freedom (DOFs). The hierarchical cascading technique (HCT) is an innovative method to reduce the calculation of DOFs by cascading a two-dimensional (2D)-FEM model of unit blocks, as reported by Koskela et al. [15,16], which significantly reduces time consumption and memory consumption on full-scale SAW devices. However, it still takes a lot of time for the researcher to build 2D-FEM models and define physical properties. Subsequently, A. Shimko et al. [17] used COMSOL software to establish a quasi-three dimensional (3D) FEM model as a unit block, and as the COMSOL software has a user-friendly interface, flexible way of modeling and mesh generation, it significantly improved the efficiency of the FEM model and physical definition. However, the computation domain of the built-in quasi-3D FEM model is a 3D block, which has much more DOFs than that of the 2D-FEM model. More recently, the GPU-assisted HCT was proposed to accelerate calculation for SAW devices with finite-length structure [18,19,20]. Although GPUs have faster data processing capabilities than CPUs and the calculation speed is further improved, the difficulty with the 2D-FEM model established by the researcher himself or the quasi-3D FEM model with more DOFs still exists, which led to a reduction of the simulation efficiency and calculation speed.

In this work, considering the finite element method model is advantageous in versatility for multi-layered and intricate structure, the partial differential equation (PDE)-based 2D-FEM model is proposed to calculate the acoustic-electric characteristics of full-scale resonators with arbitrary structure. In order to ensure its accuracy, the proposed model fully takes into account the propagation loss, dielectric loss, electrode resistance loss and electrode shape, making it consider practical factors as much as possible, and the angle of the trapezoid shape electrode was considered according to the process level. First, the PDE-based 2D-FEM model is solved by a solver of the PDE module embedded in COMSOL, and due to its user-friendly interface, flexible definition, the simulation efficiency for the researcher, it is significantly improved. Then, FEM matrices derived from COMSOL were transferred to MATLAB via LiveLink for the remaining calculation, and because the computation domain of the proposed model is a 2D plane, the DOFs are significantly less than that of the quasi-3D FEM model. It is noted that the adequate number of mesh grids and DOFs is required to ensure the accuracy of the SAW simulation. In general, a wavelength must contain at least four grids or eight nodes, which can characterize accurately the behavior of SAW devices. Considering the number of DOFs, the PDE-based 2D-FEM model has remarkable advantages over a quasi-3D or 3D FEM model. Due to the fact that the calculation speed of the FEM largely depends on the number of DOFs, with the help of GPU-assisted HCT, the PDE-based 2D-FEM model can achieve simulation effectively and efficiently for full-scale SAW devices of large size and arbitrary structure.

In addition, because the packaging structure has an increasing influence on the electrical performance of the SAW device by creating parasitic capacitance and inductance [21], the electromagnetic effect of the SAW filter is considered in the calculation process. By combining the acoustic field, electric field and electromagnetic field, hybrid full-wave simulation technology has been proven to be an efficient and powerful method to simulate the behavior of a SAW filter with higher accuracy. Firstly, the practical solid model of the SAW filter package is constructed and then the electromagnetic effect of the entire package is calculated in the High Frequency Structure Simulator (HFSS) software. Based on hybrid full-wave analysis, the frequency response of the TC-SAW filter on 5° YX-cut LiNbO_3_ substrate is investigated, and the simulated insertion loss curve is in good agreement with the measurement. The validity and accuracy of the proposed method were confirmed.

The remainder of this paper is divided into three sections. Section 2 is devoted to the PDE-based 2D-FEM model for a full-scaled resonator and the electromagnetic model for a filter. In Section 3, the admittance curve and the relative bandwidth of the TC-SAW ladder resonator and the insertion loss curve of the TC-SAW ladder filter are calculated and compared with the experimental results. Finally, conclusions are discussed in detail in the last section of the paper.

## 2. Simulation Techniques

### 2.1. Calculation Procedure

**Figure 1:**
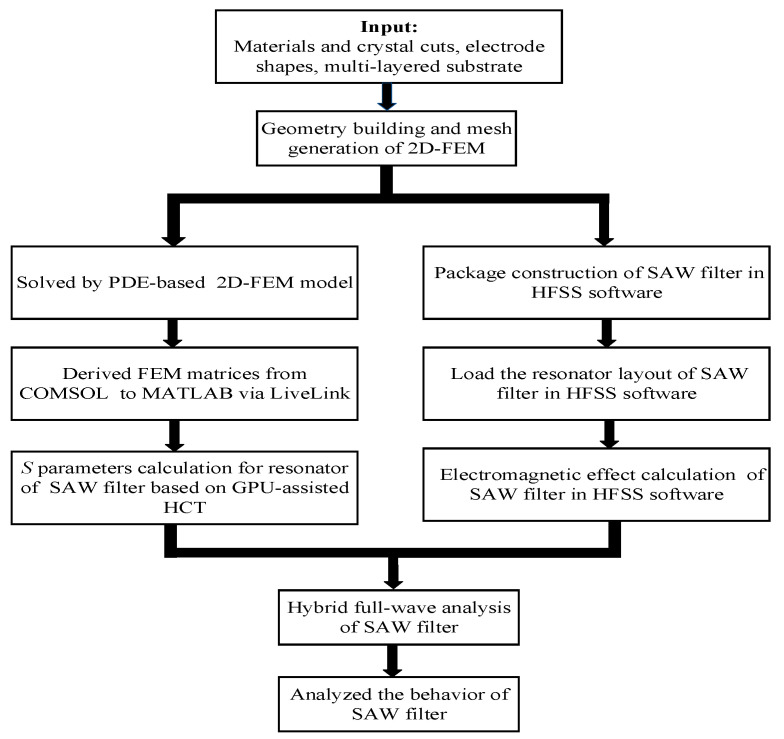
The overall flow chart of calculation for the surface acoustic wave (SAW) filter.

Figure 1 shows the overall flow chart of the calculation procedure for the SAW filter. To obtain the acoustic-electric characteristic of a SAW resonator, a PDE-based 2D-FEM model of the resonators in the SAW filter was solved by a solver of the PDE module embedded in the FEM software COMSOL. GPU-assisted hierarchical cascading technology (GPU-HCT) was used to achieve calculation acceleration. The calculation of the finite-length resonator was implemented on a workstation (Intel i9-9900K CPU @ 3.60 GHz, 96 GB RAM) with NVIDIA TITAN RTX GPUs (24 GB HBM2 memory, CUDA Compute Capability 10.0 and total of 4608 CUDA cores).

Meanwhile, the practical solid model of the RF filter package was constructed in HFSS software and the parasitic electromagnetics were calculated in the design process. Finally, simulation for the SAW filter with higher accuracy and speed was achieved by combining the acoustic behavior, electric behavior and electromagnetic behavior.

### 2.2. A PDE-based 2D-FEM Model for Resonator

**Figure 2:**
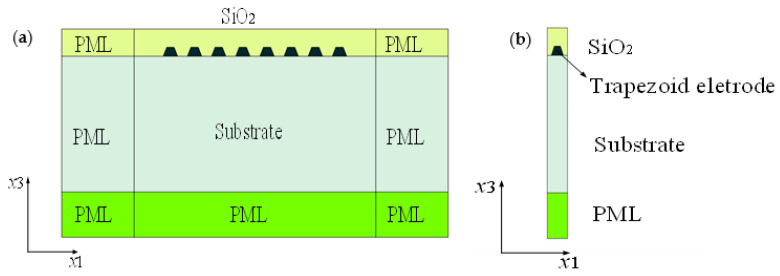
Schematic diagram of finite-length temperature compensated (TC)-SAW resonator. (**a**) The whole TC-SAW resonator; (**b**) the unit block of the TC-SAW resonator.

Without loss of generality, the acoustic surface wave was assumed to propagate in the $x_{1}$ direction and the partial derivative of the physical field along the $x_{2}$ direction ∂/∂$x_{2}\;$= 0. It means that the behavior of SAW devices can be characterized by the 2D-FEM model, which is equivalent to the previously reported quasi-3D FEM [17]. As shown in Figure 2a, the finite-length structure of the TC-SAW resonator, with the trapezoid shape electrode, is surrounded with a perfectly matched layer (PML) for absorbing thoroughly unwanted reflective waves at any incident angle, and the trapezoid angle of the electrode is set according to the practical processing technology. Figure 2b shows the unit block of TC-SAW devices.

For piezoelectric material, the relation of the mechanical displacement and electric field is described through the coupled constitutive equations [22]

(1) $${Τ}_{I}=c_{IJ}S_{J}-e_{Ij}E_{j}$$

(2) $$D_{i}=e_{iJ}S_{J}+\varepsilon_{ij}E_{j}$$

where ${Τ}_{I}$ and $S_{J}$ are stress and strain tensors, respectively. $c_{IJ}$, $e_{iJ}$ and $\varepsilon_{ij}$ are stiffness constant, piezoelectric stress constant and dielectric permittivity constant, respectively. $D_{i}$ and $E_{j}$ are the electric displacement vector and electric field, respectively.

The relation of strain and mechanical displacement can be described as

(3) $$S_{J}=\nabla_{iJ}u_{i}$$

where

(4) $$\nabla_{iJ}=\left[\begin{array}{cccccc} \frac{\partial }{\partial x_{1}} & 0 & 0 & 0 & \frac{\partial }{\partial x_{3}} & \frac{\partial }{\partial x_{2}}\\ 0 & \frac{\partial }{\partial x_{2}} & 0 & \frac{\partial }{\partial x_{3}} & 0 & \frac{\partial }{\partial x_{1}}\\ 0 & 0 & \frac{\partial }{\partial x_{3}} & \frac{\partial }{\partial x_{2}} & \frac{\partial }{\partial x_{1}} & 0 \end{array}\right]$$

According to Maxwell’s equations and boundary conditions of SAW devices, the relation among the electric displacement $D_{i}$, electric field $E_{j}$, electric potential $\phi_{j}$ and charge density $\rho_{s}$ are expressed as

(5) D=εE

(6) E=−∇ϕ

(7) ∇·D=0

(8) $$\nabla \cdot D=\rho_{s}$$

where, the Nabla operator $\nabla =\left[\begin{array}{ccc} \frac{\partial }{\partial x_{1}} & \frac{\partial }{\partial x_{2}} & \frac{\partial }{\partial x_{3}} \end{array}\right]$, Equation (7) is applied in a piezoelectric medium, and Equation (8) is applied on the interface between the piezoelectric medium and electrode.

In this work, we assume that the length of the aperture along the $x_{2}$ direction is infinite (∂/∂$x_{2}\;$= 0). Thus, the operator $\nabla_{iJ}$ and Nabla operator ∇ can be expressed as the following equation. According to Equations (9) and (10), only the $x_{1}$ and $x_{2}$ coordinate axes are contained in the operator $\nabla_{iJ}$ and the Nabla operator ∇. That is to say, the computation domain of the PDE-based 2D-FEM model is a 2D plane, as shown in Figure 2. Obviously, the DOFs are significantly less than that of the quasi-3D-FEM model reported by A. Shimko et al.

(9) $$\nabla_{iJ}=\left[\begin{array}{cccccc} \frac{\partial }{\partial x_{1}} & 0 & 0 & 0 & \frac{\partial }{\partial x_{3}} & 0\\ 0 & 0 & 0 & \frac{\partial }{\partial x_{3}} & 0 & \frac{\partial }{\partial x_{1}}\\ 0 & 0 & \frac{\partial }{\partial x_{3}} & 0 & \frac{\partial }{\partial x_{1}} & 0 \end{array}\right]$$

(10) $$\nabla =\left[\begin{array}{ccc} \frac{\partial }{\partial x_{1}} & 0 & \frac{\partial }{\partial x_{3}} \end{array}\right]$$

Assuming that there is no external force applied, the equilibrium equation in the piezoelectric medium can be described with tensor form

(11) $$\nabla_{iJ}c_{JK}\nabla_{Kl}u_{l}+\nabla_{iJ}e_{JK}\nabla \phi =\rho {\ddot{u}}_{i}$$

(12) $$-\nabla_{i}\varepsilon_{im}\nabla_{m}\phi +\nabla e_{iK}\nabla_{Kj}u_{j}=0$$

Correspondingly, the derived equilibrium Equations (11) and (12) can be converted with the matrix form as follows. Based on the 2D-FEM model, four solutions $u_{1},u_{2},u_{3}$ and ϕ of the equilibrium equation can be obtained by solving Equation (13). In addition, Voigt’s notation is used in Equation (13).

(13) $$\begin{array}{cc} \left[\begin{array}{ccc} \rho \omega^{2}u_{1} & & \\ & \rho \omega^{2}u_{2} & \\ & & \begin{array}{cc} \rho \omega^{2}u_{3} & \\ & 0 \end{array} \end{array}\right]-\nabla & \\ & \cdot [\begin{array}{cccc} \left[\begin{array}{cc} c_{11} & c_{15}\\ c_{51} & c_{55} \end{array}\right] & \left[\begin{array}{cc} c_{16} & c_{14}\\ c_{56} & c_{54} \end{array}\right] & \left[\begin{array}{cc} c_{15} & c_{13}\\ c_{55} & c_{53} \end{array}\right] & \left[\begin{array}{cc} e_{11} & e_{31}\\ e_{15} & e_{35} \end{array}\right]\\ \left[\begin{array}{cc} c_{61} & c_{65}\\ c_{41} & c_{45} \end{array}\right] & \left[\begin{array}{cc} c_{66} & c_{64}\\ c_{46} & c_{44} \end{array}\right] & \left[\begin{array}{cc} c_{66} & c_{64}\\ c_{46} & c_{44} \end{array}\right] & \left[\begin{array}{cc} e_{16} & e_{36}\\ e_{14} & e_{34} \end{array}\right]\\ \left[\begin{array}{cc} c_{51} & c_{55}\\ c_{31} & c_{35} \end{array}\right] & \left[\begin{array}{cc} c_{56} & c_{54}\\ c_{36} & c_{34} \end{array}\right] & \left[\begin{array}{cc} c_{55} & c_{53}\\ c_{35} & c_{33} \end{array}\right] & \left[\begin{array}{cc} e_{15} & e_{35}\\ e_{13} & e_{33} \end{array}\right]\\ \left[\begin{array}{cc} e_{11} & e_{15}\\ e_{31} & e_{35} \end{array}\right] & \left[\begin{array}{cc} e_{16} & e_{14}\\ e_{36} & e_{34} \end{array}\right] & \left[\begin{array}{cc} e_{15} & e_{13}\\ e_{35} & e_{33} \end{array}\right] & \left[\begin{array}{cc} -\varepsilon_{11} & -\varepsilon_{13}\\ -\varepsilon_{31} & -\varepsilon_{33} \end{array}\right] \end{array}]\left[\begin{array}{c} \nabla u_{1}\\ \nabla u_{2}\\ \begin{array}{c} \nabla u_{3}\\ \nabla \phi \end{array} \end{array}\right]=0 \end{array}$$

where ω is angular frequency, and ρ is density. The main formulas and corresponding description of PDE-based 2D-FEM model are given in Supplementary Material. For clarity and space limitations, the detailed deduction process is not mentioned in this section.

As FEM software COMSOL Multiphysics provides a mathematics module, it provides a general form PDE interface for solving piezoelectric Equation (13). In the case of four dependent variables $u_{1}$, $u_{2}$, $u_{3}$, ϕ, a general form system of the equation takes the following form (14) [23].

(14) $$e_{a}^{lk}\frac{\partial^{2}u_{k}}{\partial t^{2}}+d_{a}^{lk}\frac{\partial u_{k}}{\partial t}-\nabla \cdot \left(c\nabla u_{k}+\alpha u_{k}-\gamma \right)+\beta \nabla u_{k}+au_{k}=f_{l}\;\;\;\;\;\;\;\;in\;\Omega$$

where the equation index *l* and *k* ranges from 1 to 4, $e_{a}^{lk}$ is the mass coefficients and $d_{a}^{lk}$ is damping coefficients. *c* is the diffusion coefficient. α is the conservative flux convection coefficient. $\gamma_{l}$ is the conservative flux source term, $\beta_{l}$ is the convection coefficient. a is the absorption coefficient. $f_{l}$ is the source term, Ω is the computational domain. Note that $e_{a}^{lk}$, $d_{a}^{lk}$ and $c_{a}^{lk}$ are four-by-four matrices,$\;\gamma_{l}$, $\beta_{l}$ and $f_{l}$ are four-by-one matrices, α and a is scalar.

According to Expression (13), the coefficient of Equation (14) is given by

$$e_{a}^{lk}=\left[\begin{array}{ccc} \rho & & \\ & \rho & \\ & & \begin{array}{cc} \rho & \\ & 0 \end{array} \end{array}\right]\;c=\;\left[\begin{array}{cccc} \left[\begin{array}{cc} c_{11} & c_{15}\\ c_{51} & c_{55} \end{array}\right] & \left[\begin{array}{cc} c_{16} & c_{14}\\ c_{56} & c_{54} \end{array}\right] & \left[\begin{array}{cc} c_{15} & c_{13}\\ c_{55} & c_{53} \end{array}\right] & \left[\begin{array}{cc} e_{11} & e_{31}\\ e_{15} & e_{35} \end{array}\right]\\ \left[\begin{array}{cc} c_{61} & c_{65}\\ c_{41} & c_{45} \end{array}\right] & \left[\begin{array}{cc} c_{66} & c_{64}\\ c_{46} & c_{44} \end{array}\right] & \left[\begin{array}{cc} c_{66} & c_{64}\\ c_{46} & c_{44} \end{array}\right] & \left[\begin{array}{cc} e_{16} & e_{36}\\ e_{14} & e_{34} \end{array}\right]\\ \left[\begin{array}{cc} c_{51} & c_{55}\\ c_{31} & c_{35} \end{array}\right] & \left[\begin{array}{cc} c_{56} & c_{54}\\ c_{36} & c_{34} \end{array}\right] & \left[\begin{array}{cc} c_{55} & c_{53}\\ c_{35} & c_{33} \end{array}\right] & \left[\begin{array}{cc} e_{15} & e_{35}\\ e_{13} & e_{33} \end{array}\right]\\ \left[\begin{array}{cc} e_{11} & e_{15}\\ e_{31} & e_{35} \end{array}\right] & \left[\begin{array}{cc} e_{16} & e_{14}\\ e_{36} & e_{34} \end{array}\right] & \left[\begin{array}{cc} e_{15} & e_{13}\\ e_{35} & e_{33} \end{array}\right] & \left[\begin{array}{cc} -\varepsilon_{11} & -\varepsilon_{13}\\ -\varepsilon_{31} & -\varepsilon_{33} \end{array}\right] \end{array}\right]\;d_{a}^{lk}=0,\alpha =0,\;\gamma =0,\;\beta =0,\;a=0,\;f_{l}=0.$$

Based on the above analysis, we assume that ∂/∂$x_{2}$=0, the PDE-based 2D-FEM model is introduced in detail and four solutions $u_{1},u_{2},u_{3}$ and ϕ of Equation (13) can be obtained by solved according to Equation (14). Therefore, the PDE-based 2D-FEM model is equivalent to the built-in quasi-3D FEM model of COMSOL Multiphysics reported by A. Shimko et al. [17]. Obviously, the proposed method for SAW resonators calculation has natural advantages with fewer DOFs. In addition, to achieve quicker calculations for SAW devices, the GPU-assisted HCT [20] is employed to achieve acceleration.

### 2.3. GPU-Assisted HCT for Full-Scale Resonator Simulation

**Figure 3:**
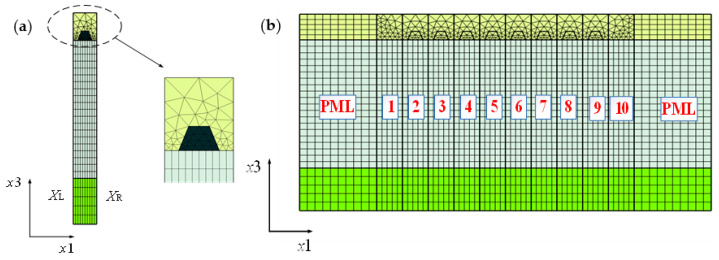
2D-finite element method (FEM) mesh of TC-SAW devices. (**a**) Mesh of unit block with trapezoid shape electrode; (**b**) the finite-length TC-SAW devices consist of a unit block.

To achieve quicker calculation for SAW devices, the GPU-assisted HCT is employed to achieve acceleration. Figure 3a shows the multi-layered FEM mesh of the TC-SAW unit block with a trapezoid electrode. This FEM model is achieved easily in COMSOL Multiphysics due to its user-friendly interface, flexible way of modeling and mesh generation, which can greatly reduce the complicated process of modeling and physical properties definition. Figure 3b shows the unit block is used to cascade into a finite-length structure by means of the continuity of the mechanical displacement and the electric potential located at the left- and right-hand edge.

According to the literature [15], linear system equations of the unit block can be written as the following Equation (15). Two ways are employed to achieve acceleration: one is to transfer A-matrices from RAM to the GPU due to the fact that GPUs have faster data processing capabilities than CPUs. The other is to eliminate the internal DOFs $X_{I}$ from the system in Equation (15) by HCT, and the system A-matrices can be greatly reduced in dimensionality.

(15) $$\left[\begin{array}{ccc} A_{LL} & A_{LI} & \begin{array}{cc} 0 & \;A_{LV} \end{array}\\ A_{IL} & A_{II} & \begin{array}{cc} A_{IR} & A_{IV} \end{array}\\ \begin{array}{c} 0\\ A_{VL} \end{array} & \begin{array}{c} A_{RI}\\ A_{VI} \end{array} & \begin{array}{cc} \begin{array}{c} A_{RR}\\ A_{VR} \end{array} & \begin{array}{c} A_{RV}\\ A_{VV} \end{array} \end{array} \end{array}\right]\left[\begin{array}{c} X_{L}\\ X_{I}\\ \begin{array}{c} X_{R}\\ v \end{array} \end{array}\right]=\left[\begin{array}{c} 0\\ 0\\ \begin{array}{c} 0\\ -q \end{array} \end{array}\right]$$

where A-matrices are the system matrix, $X_{L}$, $X_{I}$, $X_{R}$ are DOFs located on the left-hand, interior domain, and right-hand unit block, respectively, which not only contains displacement $u_{1},u_{2},u_{3}$ but also ϕ, the scalar v only contains the electric potential on the surface of the electrode, q is the net surface charge on the electrode surface.

Subsequently, eliminating the internal DOFs $X_{I}$ from A-matrices, the B-matrix only includes $X_{L}$, $X_{R}$ and v. Therefore, the B-matrix can be written as

(16) $$\left[\begin{array}{ccc} B_{11} & B_{12} & B_{13}\\ B_{21} & B_{22} & B_{23}\\ B_{31} & B_{32} & B_{33} \end{array}\right]\left[\begin{array}{c} X_{L}\\ X_{R}\\ v \end{array}\right]=\left[\begin{array}{c} 0\\ 0\\ -q \end{array}\right]$$

The expression of the currents flowing into the electrodes can be written as

(17) $$I=-i\omega \left[\left(B_{31}-B_{32}\right)X_{L}+B_{33}v\right]$$

### 2.4. The Electromagnetic Model for Filter

**Figure 4:**
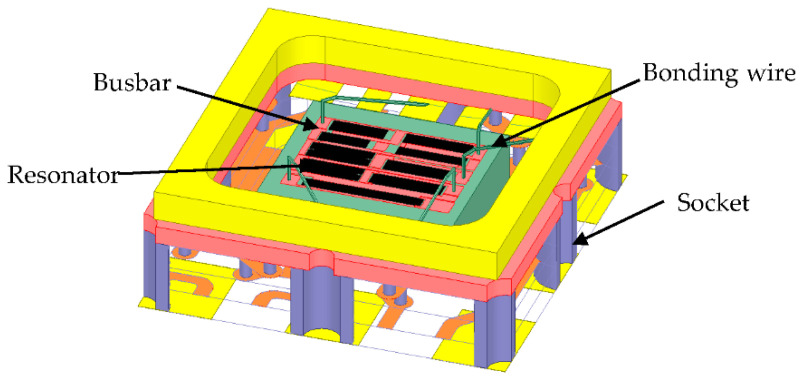
Electromagnetic model of the TC-SAW ladder filter.

With the emergence of new-type SAW devices with higher frequencies and smaller sizes, the packaging structure has an increasing influence on the electrical performance of the SAW device by creating parasitic capacitance and inductance [12,21], mainly the parasitic electromagnetics of the socket and bonding wire of the SAW device. This means the electromagnetic effect of SAW devices must be considered for more accuracy in simulation. Based on the calculated acoustic-electric characteristic of the resonator by the PDE-based 2D-FEM model, the unit block is obtainable and the practical solid model of the RF filter package is constructed in the HFSS software, and then the electromagnetic effect of the entire package is calculated in HFSS, as shown in Figure 4. Therefore, the multiphysics field of the SAW filter, containing the acoustic field, electric field and electromagnetic field, is considered in the design process and the behavior of SAW devices is analyzed with high accuracy.

## 3. Results and Discussion

### 3.1. Calculation of TC-SAW Resonator

In this work, a 2D-FEM model of a unit block of TC-SAW devices was modeled; an SH-type SAW was excited on 5° YX-cut LiNbO_3_ substrate with copper electrode, with wavelength λ = 4 µm, aperture *W* = 104 µm, 5λ thick LiNbO_3_ substrate, 3λ thick PML, metallization ratio of IDT and reflector *r* = 0.44. The fundamental material constants of the substrate were taken from Kovacs constants [24]. Correspondingly, the mesh of the 2D-FEM model was made of free triangular elements and the discretization was set as quadratic element order, and triangular elements of about 456 and DOFs of about 4004 can be obtained.

**Figure 5:**
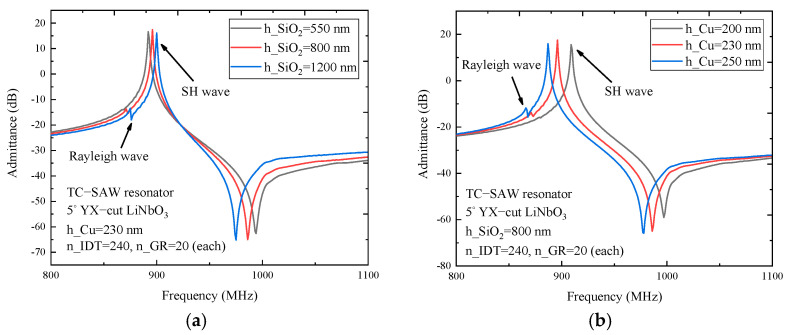
The simulated admittance curve of the TC−SAW resonator on 5° YX−cut LiNiO_3_ substrate. (**a**) The admittance Y_11_ curve with SiO_2_ thickness dependency. (**b**) The admittance Y_11_ curve with electrode thickness dependency.

Figure 5 presents the admittance curve of the TC-SAW ladder resonator in the case of different SiO_2_ thickness and electrode thickness. The results show that the resonance frequency and anti-resonance frequency have a phenomenon of frequency shift, which depend not only on SiO_2_ thickness but also on electrode thickness. As shown in Figure 5a, the admittance curve of the TC-SAW ladder resonator with a 230 nm thick copper electrode is presented for comparison, with the increase of SiO_2_ thickness, the resonance and anti-resonance frequency decreases gradually and the anti-resonance frequency decreases more. Meanwhile, the corresponding bandwidth decreases as SiO_2_ thickness increases. In addition, spurious waves occur when SiO_2_ thickness increases to 1200 nm, the reason being that the boundary conditions of the interface between the SiO_2_ coating and piezoelectric substrate were changed and the wave dispersion characteristic in the thin layer of SiO_2_ coating was also changed. 

For comparison, Figure 5b shows the admittance of the TC-SAW ladder resonator with 800 nm thick SiO_2_. As can be seen, the admittance curve moves to the left with the increase of electrode thickness. The spurious wave becomes considerably visible when electrode thickness increases to 250 nm. By comparing the differences in admittance curves affected by variation of SiO_2_ thickness and electrode thickness, it is found that the frequency response characteristic of TC-SAW devices is more sensitive to the change of electrode thickness. Therefore, the reasonable parameter selection of electrode and SiO_2_ coating is very crucial for suppressing the spurious wave when designing TC-SAW devices. 

In this analysis, the finite-length TC-SAW resonator, configured with 280 electrodes as IDT and 20 × 2 electrodes as two side reflectors, is calculated. The achieved simulation speeds of PDE-based HCT on the GPU were about 0.6 s per frequency point, which is about four times faster than that in the paper by J. Koskela [15] (with 321 electrodes need 2.4 s per frequency point). For example, the frequency ranged from 800 to 1100 MHz, the total calculational time of finite-length structure is reduced to a quarter of the previously reported method. For clarity, Table 1 shows the comparison in calculation speeds between the proposed and the previously reported methods.

**Figure 6:**
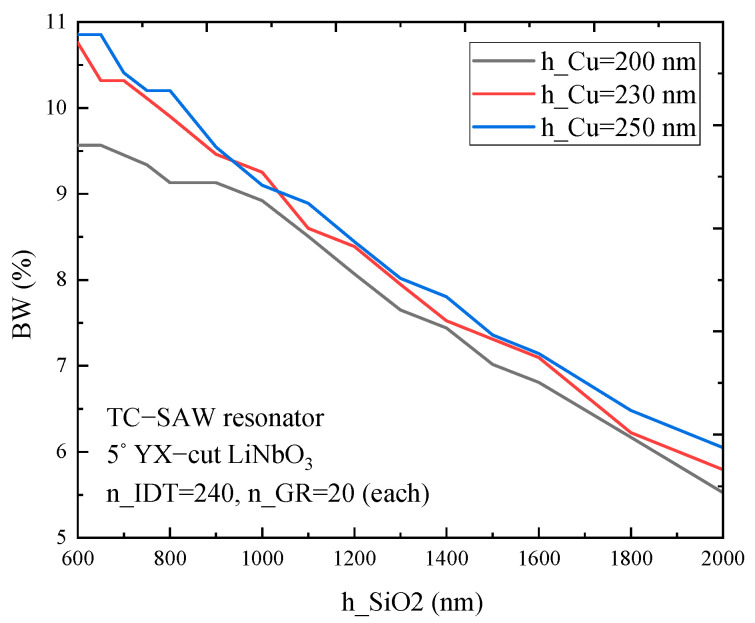
The simulated bandwidth (BW) of a TC-SAW ladder resonator on 5° YX-cut LiNbO_3_ substrate with different copper electrode and SiO_2_ thickness.

Figure 6 shows that the relative bandwidth (BW) of a one-port resonator on 5° YX-cut LiNbO_3_ substrate with a copper electrode differs with SiO_2_ thickness and electrode thickness. The results show that the relative bandwidth depends not only on SiO_2_ thickness but also on electrode thickness. Furthermore, the BW decreases gradually as SiO_2_ thickness increases and the BW decreases with the increase of electrode thickness, which provides guidance for the parameter selection of electrode and SiO_2_ coating to design the TC-SAW devices with certain BW.

### 3.2. Calculation of TC-SAW Ladder Filters

**Figure 7:**
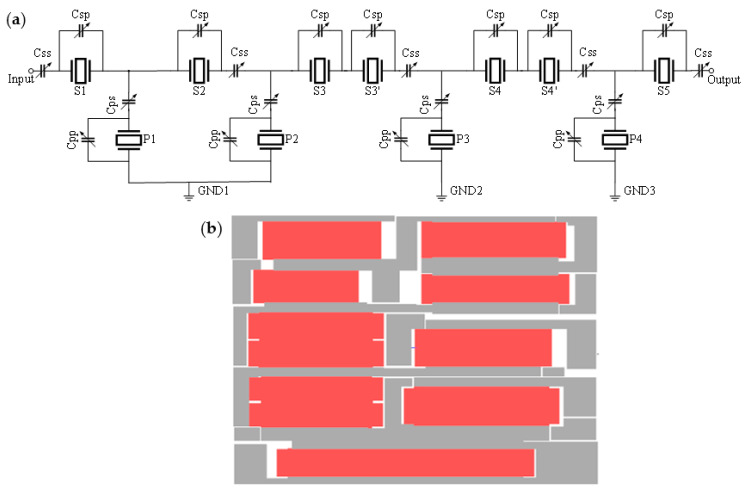
Configuration of the TC-SAW ladder filter. (**a**) Electric circuit of the TC-SAW ladder filter; (**b**) the layout of the TC-SAW ladder filter.

TC-SAW ladder filters are widely used in wireless communication systems because of good temperature stability [25]. In this case, we consider one-port ladder resonators as building blocks of ladder filters. Figure 7a shows the configuration of a TC-SAW ladder filter comprised of a T-section branch formed by series (S) and parallel (P) resonators in which the frequency characteristics determine the response of the ladder filter. In order to accurately simulate SAW devices, the corresponding layout of the TC-SAW ladder filter is designed in Figure 7b with reference to Figure 7a. The electromagnetic effect of the layout was calculated in HFSS software.

**Figure 8:**
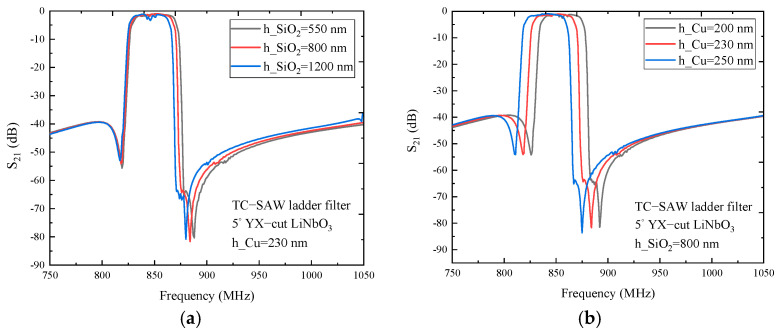
The simulated insertion loss of the TC-SAW ladder filter on 5° YX-cut LiNbO_3_ substrate. (**a**) The insertion loss S_21_ curve with SiO_2_ thickness dependency. (**b**) The insertion loss S_21_ curve with electrode thickness dependency.

Based on hybrid full-wave analysis, the frequency response characteristic of the TC-SAW ladder filter on 5° YX-cut LiNbO_3_ substrate with a copper electrode is researched within the frequency range of 750 to 1050 MHz. As shown in Figure 8a, the insertion loss curve of the TC-SAW ladder filter with the 230 nm thick electrode is presented for comparison, and the results show that the laws of the frequency response characteristic affected by SiO_2_ thickness is similar to that of the ladder resonator, and the resonance frequency and anti-resonance frequency decreases gradually with the increase of SiO_2_ thickness. The anti-frequency decreases more at the high end of the insertion loss curve. When SiO_2_ thickness increases to 1200 nm, the insertion loss becomes large at the center frequency, and the reason is that the variation of SiO_2_ thickness yields a frequency shift of the one-port ladder resonator, which leads to an impedance mismatch of the ladder filter. In Figure 8b, the insertion loss curve of the TC-SAW ladder filter with the 800 nm thick SiO_2_ is illustrated for comparison. The results show that the regularity of the frequency response characteristic affected by electrode thickness coincided with that of the ladder resonator, and the overall insertion loss curve moves to the right with the decrease of electrode thickness. When electrode thickness decreases to 200 nm, the insertion loss becomes large at center frequency due to impedance mismatch caused by a frequency shift of one-port ladder resonator. By comparing differences in frequency response curves affected by variation of electrode thickness and SiO_2_ thickness, electrode thickness has more influence on the frequency response characteristic.

### 3.3. Experimental Verification

**Figure 9:**
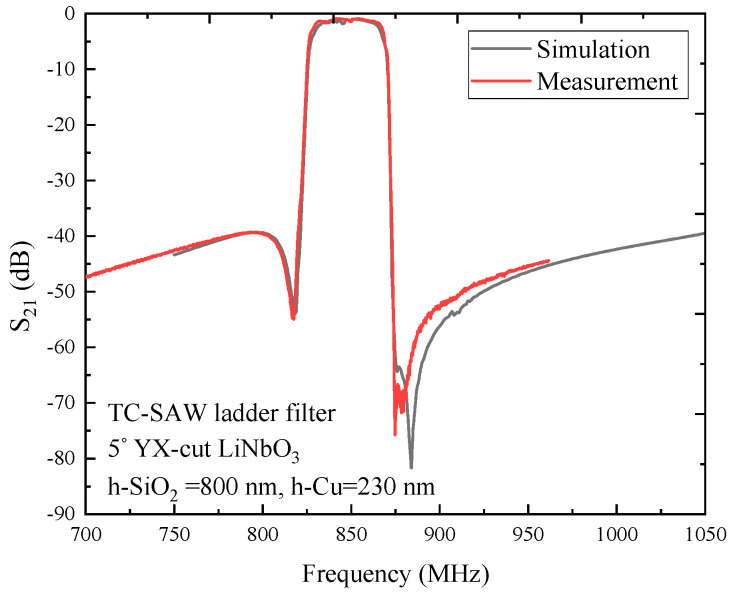
Comparison of the measurement with simulated insertion loss of the TC-SAW filter on 5° YX-cut LiNbO_3_ substrate.

To validate the simulation accuracy of the hybrid full-wave technique, a TC-SAW ladder filter on 5° YX-cut LiNbO_3_ substrate, with 800 nm thick SiO_2_ and 230 nm thick copper electrode were fabricated. The center frequency of the ladder filter was designed to be 847 MHz. By means of comparison of the simulated insertion loss curve with the measured curve, the simulated result is in good agreement with the measured result, as shown in Figure 9. The simulated curve is in good agreement with the measured result within the passband, and the small difference within the stopband is mostly due to two main reasons. First, considering the PDE-based 2D-FEM model perspective, this FEM model fully takes account of the propagation loss, dielectric loss, electrode resistance loss and electrode shape, making it consider practical factors as much as possible, but those parameters have a discrepancy with practical SAW devices. Second, from the manufacturing technology perspective, the angle of the trapezoid shape electrode was set to 7°, and in practice, the trapezoid shape electrode may be fabricated with inconsistent angles due to the instability of the process level.

## 4. Conclusions

This paper presents an effective technique for accurate and fast simulation of SAW devices based on hybrid full-wave analysis. First, the PDE-based 2D-FEM model was proposed to calculate the acoustic-electric characteristic of SAW devices, and the practical solid model of the entire package was constructed and its parasitic electromagnetic effect was calculated in HFSS software. With the use of the PDE-based 2D-FEM model to overcome the problem of a large number of DOFs, with the help of GPU-assisted HCT, the simulation for a SAW filter with higher speed and accuracy was achieved in the frequency range. The validity and accuracy of the proposed method were verified by comparing the simulated frequency response characteristics with that of the measurement of the TC-SAW ladder filter on 5° YX-cut LiNbO_3_ substrate. Considering the generality of FEM, in future work, the proposed hybrid full-wave technique can be extended to accurately analyze the key factors affecting the performance (rectangularity, bandwidth, flatness in passband) of SAW devices with complex structures, and to find out the corresponding relation between the performance and structure parameters, which is quite useful for the development of new-type SAW devices with high performance to meet the stringent requirements of RF devices.

## Figures and Tables

**Table 1:** The comparison of other methods for SAW resonator simulation. T1 donate calculation speed per frequency point, T2 donate calculation speed within the range of *f* = 800 MHz–1100 MHz.

	Regular Resonator (Reference [9])	TC-SAW Resonator (This Work)
Number of electrode	321	320
Calculation time T1	2.4 s	0.6 s
Calculation time T2	720 s	180 s

## Data Availability

The authors have access to all the data in the study (for original research articles) and accept responsibility for its validity.

## Supplementary Materials

The following are available online at https://www.mdpi.com/2072-666X/12/1/5/s1, title: Detailed derivation of the PDE model.
