# Supplementary material for: Hybrid Full-Wave Analysis of Surface Acoustic Wave Devices for Accuracy and Fast Performance Prediction
Source: Micromachines (Basel). 2020 Dec 22;12(1):5. doi: 10.3390/mi12010005 (PMC7838811; doi:10.3390/mi12010005)
Supplement: Supplementary file 1 [file micromachines-12-00005-s001.pdf]

Detailed derivation of the PDE model

1 Govern equation

In a piezoelectric substrate, the relationship, strain vs stress and displacement vs electric field, is considered linear, thus the coupled constitutive equations in piezoelectric substrate can be expressed

$$T_I = c_{IJ}S_J - e_{Ij}E_j \tag{1}$$

$$D_i = e_{iJ}S_J + \varepsilon_{ij}E_j \tag{2}$$

According to Equation (1) and Equation (2), the stress and electric displacement can be takenthe following form Equation (3) and Equation (4), respectively.

$$\begin{bmatrix} T_1 \\ T_2 \\ T_3 \\ T_4 \\ T_5 \\ T_6 \end{bmatrix} = \begin{bmatrix} c_{11} & c_{12} & c_{13} & c_{14} & c_{15} & c_{16} \\ c_{21} & c_{22} & c_{23} & c_{24} & c_{25} & c_{26} \\ c_{31} & c_{32} & c_{33} & c_{34} & c_{35} & c_{36} \\ c_{41} & c_{42} & c_{43} & c_{44} & c_{45} & c_{46} \\ c_{51} & c_{52} & c_{53} & c_{54} & c_{55} & c_{56} \\ c_{61} & c_{62} & c_{63} & c_{64} & c_{65} & c_{66} \end{bmatrix} \begin{bmatrix} S_1 \\ S_2 \\ S_3 \\ S_4 \\ S_5 \\ S_6 \end{bmatrix} - \begin{bmatrix} e_{11} & e_{21} & e_{31} \\ e_{12} & e_{22} & e_{32} \\ e_{13} & e_{23} & e_{33} \\ e_{14} & e_{24} & e_{34} \\ e_{15} & e_{25} & e_{35} \\ e_{16} & e_{26} & e_{36} \end{bmatrix} \begin{bmatrix} E_1 \\ E_2 \\ E_3 \end{bmatrix}, \tag{3}$$

$$\begin{bmatrix} D_1 \\ D_2 \\ D_3 \end{bmatrix} = \begin{bmatrix} \varepsilon_{11} & \varepsilon_{12} & \varepsilon_{13} \\ \varepsilon_{21} & \varepsilon_{22} & \varepsilon_{23} \\ \varepsilon_{31} & \varepsilon_{32} & \varepsilon_{33} \end{bmatrix} \begin{bmatrix} E_1 \\ E_2 \\ E_3 \end{bmatrix} + \begin{bmatrix} e_{11} & e_{12} & e_{13} & e_{14} & e_{15} & e_{16} \\ e_{21} & e_{22} & e_{23} & e_{24} & e_{25} & e_{26} \\ e_{31} & e_{32} & e_{33} & e_{34} & e_{35} & e_{36} \end{bmatrix} \begin{bmatrix} S_1 \\ S_2 \\ S_3 \\ S_4 \\ S_5 \\ S_6 \end{bmatrix}, \tag{4}$$

The relation of strain and mechanical displacement can be described as

$$S_J = \nabla_{iJ}u_i \tag{5}$$

where, ∇_{iJ} is the operator

$$\nabla_{iJ} = \begin{bmatrix} \frac{\partial}{\partial x_1} & 0 & 0 & 0 & \frac{\partial}{\partial x_3} & \frac{\partial}{\partial x_2} \\ 0 & \frac{\partial}{\partial x_2} & 0 & \frac{\partial}{\partial x_3} & 0 & \frac{\partial}{\partial x_1} \\ 0 & 0 & \frac{\partial}{\partial x_3} & \frac{\partial}{\partial x_2} & \frac{\partial}{\partial x_1} & 0 \end{bmatrix}, \tag{6}$$

Substituting (6) into (5), strain tensor S can be expressed as

$$S = \begin{bmatrix} S_1 \\ S_2 \\ S_3 \\ S_4 \\ S_5 \\ S_6 \end{bmatrix} = \begin{bmatrix} \frac{\partial}{\partial x_1} & 0 & 0 \\ 0 & \frac{\partial}{\partial x_2} & 0 \\ 0 & 0 & \frac{\partial}{\partial x_3} \\ 0 & \frac{\partial}{\partial x_3} & \frac{\partial}{\partial x2} \\ \frac{\partial}{\partial x_3} & 0 & \frac{\partial}{\partial x_1} \\ \frac{\partial}{\partial x_2} & \frac{\partial}{\partial x_1} & 0 \end{bmatrix} \begin{bmatrix} u_1 \\ u_2 \\ u_3 \end{bmatrix}, \tag{7}$$

Substituting (7) into (3) and (4) yields

$$\begin{bmatrix} T_1 \\ T_2 \\ T_3 \\ T_4 \\ T_5 \\ T_6 \end{bmatrix} = \begin{bmatrix} c_{11} & c_{12} & c_{13} & c_{14} & c_{15} & c_{16} \\ c_{21} & c_{22} & c_{23} & c_{24} & c_{25} & c_{26} \\ c_{31} & c_{32} & c_{33} & c_{34} & c_{35} & c_{36} \\ c_{41} & c_{42} & c_{43} & c_{44} & c_{45} & c_{46} \\ c_{51} & c_{52} & c_{53} & c_{54} & c_{55} & c_{56} \\ c_{61} & c_{62} & c_{63} & c_{64} & c_{65} & c_{66} \end{bmatrix} \begin{bmatrix} \frac{\partial}{\partial x_1} & 0 & 0 \\ 0 & \frac{\partial}{\partial x_2} & 0 \\ 0 & 0 & \frac{\partial}{\partial x_3} \\ 0 & \frac{\partial}{\partial x_3} & \frac{\partial}{\partial x2} \\ \frac{\partial}{\partial x_3} & 0 & \frac{\partial}{\partial x_1} \\ \frac{\partial}{\partial x_2} & \frac{\partial}{\partial x_1} & 0 \end{bmatrix} \begin{bmatrix} u_1 \\ u_2 \\ u_3 \end{bmatrix} - \begin{bmatrix} e_{11} & e_{21} & e_{31} \\ e_{12} & e_{22} & e_{32} \\ e_{13} & e_{23} & e_{33} \\ e_{14} & e_{24} & e_{34} \\ e_{15} & e_{25} & e_{35} \\ e_{16} & e_{26} & e_{36} \end{bmatrix} \begin{bmatrix} E_1 \\ E_2 \\ E_3 \end{bmatrix}, \tag{8}$$

$$\begin{bmatrix} D_1 \\ D_2 \\ D_3 \end{bmatrix} = \begin{bmatrix} \varepsilon_{11} & \varepsilon_{12} & \varepsilon_{13} \\ \varepsilon_{21} & \varepsilon_{22} & \varepsilon_{23} \\ \varepsilon_{31} & \varepsilon_{32} & \varepsilon_{33} \end{bmatrix} \begin{bmatrix} E_1 \\ E_2 \\ E_3 \end{bmatrix} + \begin{bmatrix} e_{11} & e_{12} & e_{13} & e_{14} & e_{15} & e_{16} \\ e_{21} & e_{22} & e_{23} & e_{24} & e_{25} & e_{26} \\ e_{31} & e_{32} & e_{33} & e_{34} & e_{35} & e_{36} \end{bmatrix} \begin{bmatrix} \frac{\partial}{\partial x_1} & 0 & 0 \\ 0 & \frac{\partial}{\partial x_2} & 0 \\ 0 & 0 & \frac{\partial}{\partial x_3} \\ 0 & \frac{\partial}{\partial x_3} & \frac{\partial}{\partial x2} \\ \frac{\partial}{\partial x_3} & 0 & \frac{\partial}{\partial x_1} \\ \frac{\partial}{\partial x_2} & \frac{\partial}{\partial x_1} & 0 \end{bmatrix} \begin{bmatrix} u_1 \\ u_2 \\ u_3 \end{bmatrix}, \tag{9}$$

2 The equilibrium equation of piezoelectric

According to the Newton Law, the equilibrium equation in piezoelectric medium is defined by

$$\nabla_{iK} T_K = \rho \frac{\partial^2 \boldsymbol{u_i}}{\partial t^2}, \tag{10}$$

According to the Maxwell's equations and electric boundary conditions of SAW devices, the relation among the electric displacement D_i, electric field E_k, electric potential ϕ_k and charge density ρ_s are defined by

$$D = \varepsilon E, \tag{11}$$
$$E = -\nabla \phi, \tag{12}$$
$$\nabla \cdot D = 0, \tag{13}$$
$$\nabla \cdot D = \rho_s, \tag{14}$$

where, $\nabla = \begin{bmatrix} \frac{\partial}{\partial x_1} & \frac{\partial}{\partial x_2} & \frac{\partial}{\partial x_3} \end{bmatrix}$, Equation (13) is applied in piezoelectric medium, Equation (14) is applied on interface between piezoelectric medium and electrode.

Assuming that there is no external force applied, the equilibrium equation of the piezoelectric relation can be described with tensor form

$$\nabla_{iJ} c_{JK} \nabla_{Kl} u_l + \nabla_{iJ} e_{JK} \nabla \phi = \rho \ddot{u}_i \tag{15}$$
$$-\nabla_i \varepsilon_{im} \nabla_m \phi + \nabla e_{iK} \nabla_{Kj} u_j = 0 \tag{16}$$

The Equation (15) and Equation (16) can be expressed with matrix form, respectively. Thus the

equilibrium equation can be expressed as

$$\begin{bmatrix} \frac{\partial}{\partial x_1} & 0 & 0 & 0 & \frac{\partial}{\partial x_3} & \frac{\partial}{\partial x_2} \\ 0 & \frac{\partial}{\partial x_2} & 0 & \frac{\partial}{\partial x_3} & 0 & \frac{\partial}{\partial x_1} \\ 0 & 0 & \frac{\partial}{\partial x_3} & \frac{\partial}{\partial x_2} & \frac{\partial}{\partial x_1} & 0 \end{bmatrix} \begin{bmatrix} c_{11} & c_{12} & c_{13} & c_{14} & c_{15} & c_{16} \\ c_{21} & c_{22} & c_{23} & c_{24} & c_{25} & c_{26} \\ c_{31} & c_{32} & c_{33} & c_{34} & c_{35} & c_{36} \\ c_{41} & c_{42} & c_{43} & c_{44} & c_{45} & c_{46} \\ c_{51} & c_{52} & c_{53} & c_{54} & c_{55} & c_{56} \\ c_{61} & c_{62} & c_{63} & c_{64} & c_{65} & c_{66} \end{bmatrix} \begin{bmatrix} \frac{\partial}{\partial x_1} & 0 & 0 \\ 0 & \frac{\partial}{\partial x_2} & 0 \\ 0 & 0 & \frac{\partial}{\partial x_3} \\ 0 & \frac{\partial}{\partial x_3} & \frac{\partial}{\partial x2} \\ \frac{\partial}{\partial x_3} & 0 & \frac{\partial}{\partial x_1} \\ \frac{\partial}{\partial x_2} & \frac{\partial}{\partial x_1} & 0 \end{bmatrix} \begin{bmatrix} u_1 \\ u_2 \\ u_3 \end{bmatrix} +$$

$$\begin{bmatrix} \frac{\partial}{\partial x_1} & 0 & 0 & 0 & \frac{\partial}{\partial x_3} & \frac{\partial}{\partial x_2} \\ 0 & \frac{\partial}{\partial x_2} & 0 & \frac{\partial}{\partial x_3} & 0 & \frac{\partial}{\partial x_1} \\ 0 & 0 & \frac{\partial}{\partial x_3} & \frac{\partial}{\partial x_2} & \frac{\partial}{\partial x_1} & 0 \end{bmatrix} \begin{bmatrix} e_{11} & e_{21} & e_{31} \\ e_{12} & e_{22} & e_{32} \\ e_{13} & e_{23} & e_{33} \\ e_{14} & e_{24} & e_{34} \\ e_{15} & e_{25} & e_{35} \\ e_{16} & e_{26} & e_{36} \end{bmatrix} \begin{bmatrix} \frac{\partial \phi}{\partial x_1} \\ \frac{\partial \phi}{\partial x_2} \\ \frac{\partial \phi}{\partial x_3} \end{bmatrix} = \begin{bmatrix} \rho \frac{\partial^2 u_1}{\partial t^2} \\ \rho \frac{\partial^2 u_2}{\partial t^2} \\ \rho \frac{\partial^2 u_3}{\partial t^2} \end{bmatrix}, \tag{17}$$

$$-\begin{bmatrix} \frac{\partial}{\partial x_1} & \frac{\partial}{\partial x_2} & \frac{\partial}{\partial x_3} \end{bmatrix} \begin{bmatrix} \varepsilon_{11} & \varepsilon_{12} & \varepsilon_{13} \\ \varepsilon_{21} & \varepsilon_{22} & \varepsilon_{23} \\ \varepsilon_{31} & \varepsilon_{32} & \varepsilon_{33} \end{bmatrix} \begin{bmatrix} \frac{\partial \phi}{\partial x_1} \\ \frac{\partial \phi}{\partial x_2} \\ \frac{\partial \phi}{\partial x_3} \end{bmatrix}$$

$$+\begin{bmatrix} \frac{\partial}{\partial x_1} & \frac{\partial}{\partial x_2} & \frac{\partial}{\partial x_3} \end{bmatrix} \begin{bmatrix} e_{11} & e_{12} & e_{13} & e_{14} & e_{15} & e_{16} \\ e_{21} & e_{22} & e_{23} & e_{24} & e_{25} & e_{26} \\ e_{31} & e_{32} & e_{33} & e_{34} & e_{35} & e_{36} \end{bmatrix} \begin{bmatrix} \frac{\partial}{\partial x_1} & 0 & 0 \\ 0 & \frac{\partial}{\partial x_2} & 0 \\ 0 & 0 & \frac{\partial}{\partial x_3} \\ 0 & \frac{\partial}{\partial x_3} & \frac{\partial}{\partial x2} \\ \frac{\partial}{\partial x_3} & 0 & \frac{\partial}{\partial x_1} \\ \frac{\partial}{\partial x_2} & \frac{\partial}{\partial x_1} & 0 \end{bmatrix} \begin{bmatrix} u_1 \\ u_2 \\ u_3 \end{bmatrix} = 0, \tag{18}$$

In this work, we assume that the partial derivative of physical field along the aperture direction is set to zero ($\partial/\partial x_2$=0), which can describe the infinite length of the aperture along x_2 direction. Thus the Equation (17) and Equation (18) can be expressed as

$$\begin{bmatrix} \frac{\partial}{\partial x_1} & 0 & 0 & 0 & \frac{\partial}{\partial x_3} & 0 \\ 0 & 0 & 0 & \frac{\partial}{\partial x_3} & 0 & \frac{\partial}{\partial x_1} \\ 0 & 0 & \frac{\partial}{\partial x_3} & 0 & \frac{\partial}{\partial x_1} & 0 \end{bmatrix} \begin{bmatrix} c_{11} & c_{12} & c_{13} & c_{14} & c_{15} & c_{16} \\ c_{21} & c_{22} & c_{23} & c_{24} & c_{25} & c_{26} \\ c_{31} & c_{32} & c_{33} & c_{34} & c_{35} & c_{36} \\ c_{41} & c_{42} & c_{43} & c_{44} & c_{45} & c_{46} \\ c_{51} & c_{52} & c_{53} & c_{54} & c_{55} & c_{56} \\ c_{61} & c_{62} & c_{63} & c_{64} & c_{65} & c_{66} \end{bmatrix} \begin{bmatrix} \frac{\partial}{\partial x_1} & 0 & 0 \\ 0 & 0 & 0 \\ 0 & 0 & \frac{\partial}{\partial x_3} \\ 0 & \frac{\partial}{\partial x_3} & 0 \\ \frac{\partial}{\partial x_3} & 0 & \frac{\partial}{\partial x_1} \\ 0 & \frac{\partial}{\partial x_1} & 0 \end{bmatrix} \begin{bmatrix} u_1 \\ u_2 \\ u_3 \end{bmatrix} +$$

$$\begin{bmatrix} \frac{\partial}{\partial x_1} & 0 & 0 & 0 & \frac{\partial}{\partial x_3} & 0 \\ 0 & 0 & 0 & \frac{\partial}{\partial x_3} & 0 & \frac{\partial}{\partial x_1} \\ 0 & 0 & \frac{\partial}{\partial x_3} & 0 & \frac{\partial}{\partial x_1} & 0 \end{bmatrix} \begin{bmatrix} e_{11} & e_{21} & e_{31} \\ e_{12} & e_{22} & e_{32} \\ e_{13} & e_{23} & e_{33} \\ e_{14} & e_{24} & e_{34} \\ e_{15} & e_{25} & e_{35} \\ e_{16} & e_{26} & e_{36} \end{bmatrix} \begin{bmatrix} \frac{\partial \phi}{\partial x_1} \\ 0 \\ \frac{\partial \phi}{\partial x_3} \end{bmatrix} = \begin{bmatrix} \rho \frac{\partial^2 u_1}{\partial t^2} \\ \rho \frac{\partial^2 u_2}{\partial t^2} \\ \rho \frac{\partial^2 u_3}{\partial t^2} \end{bmatrix},$$

(19)

$$-\begin{bmatrix} \frac{\partial}{\partial x_1} & 0 & \frac{\partial}{\partial x_3} \end{bmatrix} \begin{bmatrix} \varepsilon_{11} & \varepsilon_{12} & \varepsilon_{13} \\ \varepsilon_{21} & \varepsilon_{22} & \varepsilon_{23} \\ \varepsilon_{31} & \varepsilon_{32} & \varepsilon_{33} \end{bmatrix} \begin{bmatrix} \frac{\partial \phi}{\partial x_1} \\ 0 \\ \frac{\partial \phi}{\partial x_3} \end{bmatrix}$$

$$+\begin{bmatrix} \frac{\partial}{\partial x_1} & 0 & \frac{\partial}{\partial x_3} \end{bmatrix} \begin{bmatrix} e_{11} & e_{12} & e_{13} & e_{14} & e_{15} & e_{16} \\ e_{21} & e_{22} & e_{23} & e_{24} & e_{25} & e_{26} \\ e_{31} & e_{32} & e_{33} & e_{34} & e_{35} & e_{36} \end{bmatrix} \begin{bmatrix} \frac{\partial}{\partial x_1} & 0 & 0 \\ 0 & 0 & 0 \\ 0 & 0 & \frac{\partial}{\partial x_3} \\ 0 & \frac{\partial}{\partial x_3} & 0 \\ \frac{\partial}{\partial x_3} & 0 & \frac{\partial}{\partial x_1} \\ 0 & \frac{\partial}{\partial x_1} & 0 \end{bmatrix} \begin{bmatrix} u_1 \\ u_2 \\ u_3 \end{bmatrix} = 0,$$

(20)

According to Equation (19) and Equation (20), only the x_1 and x_2 coordinate axes are contained in the equilibrium equation for describing the infinite length along x_2 direction. That's to say, this derived PDE-based 2D-FEM model is equivalent to built-in quasi-3D FEM model previously reported by Shimko et al. [24]. Therefore, only 2D-FEM model of the single-finger established by COMSOL can obtain four solutions u_1, u_2, u_3 and ϕ of the Equation (19) and Equation (20).

3 The solution of PDEs by COMSOL

Due to FEM software COMSOL Multiphysics provides a mathematics module, and its built-in PDE interface supports coefficient form PDE as (21)

$$e_a^{lk} \frac{\partial^2 u_k}{\partial t^2} + d_a^{lk} \frac{\partial u_k}{\partial t} - \nabla \cdot (c \nabla u_k + \alpha u_k - \gamma) + \beta \nabla u_k + a u_k = f_l \qquad in\, \Omega,$$

(21)

The Equation (21) enables solving PDE Equation (19) and Equation (20) with multiple dependent variables. Correspondingly, according to the form of Equation (21), the piezoelectric equilibrium equation Equation (19) and Equation (20) can be converted as follows

$$\begin{bmatrix} \rho\omega^2 u_1 & & & \\ & \rho\omega^2 u_2 & & \\ & & \rho\omega^2 u_3 & \\ & & & 0 \end{bmatrix} = \nabla \cdot \begin{bmatrix} \begin{bmatrix} c_{11} & c_{15} \\ c_{51} & c_{55} \end{bmatrix} & \begin{bmatrix} c_{16} & c_{14} \\ c_{56} & c_{54} \end{bmatrix} & \begin{bmatrix} c_{15} & c_{13} \\ c_{55} & c_{53} \end{bmatrix} & \begin{bmatrix} e_{11} & e_{31} \\ e_{15} & e_{35} \end{bmatrix} \\ \begin{bmatrix} c_{61} & c_{65} \\ c_{41} & c_{45} \end{bmatrix} & \begin{bmatrix} c_{66} & c_{64} \\ c_{46} & c_{44} \end{bmatrix} & \begin{bmatrix} c_{65} & c_{63} \\ c_{45} & c_{43} \end{bmatrix} & \begin{bmatrix} e_{16} & e_{36} \\ e_{14} & e_{34} \end{bmatrix} \\ \begin{bmatrix} c_{51} & c_{55} \\ c_{31} & c_{35} \end{bmatrix} & \begin{bmatrix} c_{56} & c_{54} \\ c_{36} & c_{34} \end{bmatrix} & \begin{bmatrix} c_{55} & c_{53} \\ c_{35} & c_{33} \end{bmatrix} & \begin{bmatrix} e_{15} & e_{35} \\ e_{13} & e_{33} \end{bmatrix} \\ \begin{bmatrix} e_{11} & e_{15} \\ e_{31} & e_{35} \end{bmatrix} & \begin{bmatrix} e_{16} & e_{14} \\ e_{36} & e_{34} \end{bmatrix} & \begin{bmatrix} e_{15} & e_{13} \\ e_{35} & e_{33} \end{bmatrix} & \begin{bmatrix} -\varepsilon_{11} & -\varepsilon_{13} \\ -\varepsilon_{31} & -\varepsilon_{33} \end{bmatrix} \end{bmatrix} \begin{bmatrix} \nabla u_1 \\ \nabla u_2 \\ \nabla u_3 \\ \nabla \phi \end{bmatrix},$$

(22)

According to the expression (22), the coefficient of expression (21) is given by

$$e_a^{lk} = \begin{bmatrix} \rho & & & \\ & \rho & & \\ & & \rho & \\ & & & 0 \end{bmatrix}, \tag{23}$$

$$c = \begin{bmatrix} \begin{bmatrix} c_{11} & c_{15} \\ c_{51} & c_{55} \end{bmatrix} & \begin{bmatrix} c_{16} & c_{14} \\ c_{56} & c_{54} \end{bmatrix} & \begin{bmatrix} c_{15} & c_{13} \\ c_{55} & c_{53} \end{bmatrix} & \begin{bmatrix} e_{11} & e_{31} \\ e_{15} & e_{35} \end{bmatrix} \\ \begin{bmatrix} c_{61} & c_{65} \\ c_{41} & c_{45} \end{bmatrix} & \begin{bmatrix} c_{66} & c_{64} \\ c_{46} & c_{44} \end{bmatrix} & \begin{bmatrix} c_{65} & c_{63} \\ c_{45} & c_{43} \end{bmatrix} & \begin{bmatrix} e_{16} & e_{36} \\ e_{14} & e_{34} \end{bmatrix} \\ \begin{bmatrix} c_{51} & c_{55} \\ c_{31} & c_{35} \end{bmatrix} & \begin{bmatrix} c_{56} & c_{54} \\ c_{36} & c_{34} \end{bmatrix} & \begin{bmatrix} c_{55} & c_{53} \\ c_{35} & c_{33} \end{bmatrix} & \begin{bmatrix} e_{15} & e_{35} \\ e_{13} & e_{33} \end{bmatrix} \\ \begin{bmatrix} e_{11} & e_{15} \\ e_{31} & e_{35} \end{bmatrix} & \begin{bmatrix} e_{16} & e_{14} \\ e_{36} & e_{34} \end{bmatrix} & \begin{bmatrix} e_{15} & e_{13} \\ e_{35} & e_{33} \end{bmatrix} & \begin{bmatrix} -\varepsilon_{11} & -\varepsilon_{13} \\ -\varepsilon_{31} & -\varepsilon_{33} \end{bmatrix} \end{bmatrix}, \tag{24}$$

$$d_a^{lk} = 0, \tag{25}$$

$$\alpha = 0, \tag{26}$$

$$\gamma = 0, \tag{27}$$

$$\beta = 0, \tag{28}$$

$$\mathrm{a} = 0, \tag{29}$$

$$f_l = 0, \tag{30}$$
